# Efficacy and tolerability of gemtuzumab ozogamicin (anti-CD33 monoclonal antibody, CMA-676, Mylotarg^®^) in children with relapsed/refractory myeloid leukemia

**DOI:** 10.1186/1471-2407-6-172

**Published:** 2006-06-28

**Authors:** Benoit Brethon, Anne Auvrignon, Claire Galambrun, Karima Yakouben, Thierry Leblanc, Yves Bertrand, Guy Leverger, André Baruchel

**Affiliations:** 1 Unité de Pédiatrie à Orientation Hématologique, Hôpital Saint-Louis, Paris, France; 2 Unité d'Onco-Hématologie Pédiatrique, Hôpital d'Enfants Armand Trousseau, Paris, France; 3 Unité d'Immuno-Hématologie Pédiatrique et Transplantation de Moelle Osseuse, Hôpital Debrousse, Lyon, France; 4 Unité d'Hématologie Pédiatrique, Hôpital Robert Debré, Paris, France

## Abstract

**Background:**

Gemtuzumab ozogamicin (GO) is a cytotoxic anti-CD33 monoclonal antibody that has given promising preliminary results in adult myeloid CD33+ AML. We conducted a retrospective multicenter study of 12 children treated with GO on a compassionate basis (median age 5.5 y). Three patients (2 MDS/AML, 1 JMML) were refractory to first-line treatment, 8 patients with de novo AML were in refractory first relapse, and one patient with de novo AML was in 2^nd ^relapse after stem cell transplantation (SCT). CD33 expression exceeded 20% in all cases.

**Methods:**

GO was administered alone, at a unit dose of 3–9 mg/m^2^, once (3 patients), twice (3 patients), three (5 patients) or five times (1 patient). Mean follow-up was 128 days (8–585 d).

**Results:**

There were three complete responses (25%) leading to further curative treatment (SCT). Treatment failed in the other nine patients, and only one patient was alive at the end of follow-up. NCI-CTC grade III/IV adverse events comprised hematological toxicity (n = 12), hypertransaminasemia (n = 2), allergy and hyperbilirubinemia (1 case each). There was only one major adverse event (grade IV allergy). No case of sinusoidal obstruction syndrome occurred.

**Conclusion:**

These results warrant a prospective trial of GO in a larger population of children with AML.

## Background

Despite gradual improvements over the years, the survival rate among children with acute myeloblastic leukemia (AML) was only 50 to 60% during the last decade [1-6]. About 10% of children do not enter first complete remission (CR). In addition, second CR is often difficult to achieve, even with high-dose cytarabine. Patients who relapse therefore have few therapeutic options. New cytotoxic agents, including nucleoside analogs, are currently being evaluated [2,7-9].

An alternative to chemotherapy (CT) is to target leukemic blasts with monoclonal antibodies. Approximately 80% to 90% of pediatric AML patients have myeloid blast cells that express the CD33 surface antigen [10]. This antigen is present on normal hematopoietic progenitor cells but not on normal hematopoietic stem cells or on non hematopoietic cells [11]. Gemtuzumab ozogamicin (GO) is an immunoconjugate consisting of a humanized anti-CD33 IgG_4κ _antibody linked to the cytotoxic compound N-acetyl-γ-calicheamicin dimethylhydrazine, a member of the enediyne antitumor antibiotic family [12,13]. GO selectively targets CD33+ cells and was specifically developed for the treatment of AML. After receptor binding, the complex is rapidly internalized and calicheamicin is released intracellularly. Calicheamicins are known for their extreme cytotoxic potency, attributed to double-stranded DNA cleavage at specific sequences [14,15]. In phase I/II studies, approximately 30% of adults with relapsed AML responded to GO [12,13,16]. Severe myelosuppression is common, however, and platelet recovery can be slow, probably owing to CD33-expressing platelet precursor damage [13]. Toxicity is relatively mild compared with classical multiagent CT, especially with regard to mucositis and infections [17], but GO can cause severe liver toxicity in the form of a sinusoidal obstruction syndrome (SOS) [18,19] Several factors can increase the risk of hepatotoxicity, including previous stem cell transplantation (SCT) [20]. Prior exposure to GO is also known to increase the risk of SOS in patients who subsequently undergo myeloablative SCT [21] GO has been approved in the United States for the treatment of elderly patients with relapsed AML [16].

GO has rarely been used in children. Sievers et al reported preliminary results of a phase I ascending-dose study of GO in 18 children with relapsed or refractory AML [22]. Likewise, Zwaan et al used GO (up to three doses) to treat 15 children with relapsed/refractory CD33+ AML, on a compassionate-use basis [23,24]. More recently Arceci et al reported a dose-escalation study of 29 children with multiple relapsed or primary refractory AML [25]. Here we report our experience with GO monotherapy in 12 children with relapsed or refractory AML.

## Methods

This retrospective study involved 12 children treated with GO between March 1999 and April 2004 on a compassionate-use basis in four pediatric centers. GO therapy was approved by the french agency for health product safety (AFSSAPS), and the guardians' and/or patients' informed consent was obtained. The cutoff date for this analysis was 30 September 2004. The children had myelodysplasia (MDS)/AML refractory to standard induction therapy (n = 2), juvenile myelomonocytic leukemia (JMML) refractory to several cytotoxic drugs and retinoic acid (n = 1), first relapse of AML refractory to reinduction therapy (n = 8), or AML in second relapse after SCT (n = 1).

The patients' characteristics at initial diagnosis are shown in Table 1. Median age was 5.5 years (1.0–17.2 y), and there were 7 boys and 5 girls. The initial diagnoses were de novo AML in 9 cases (M1 = 1; M2 = 2; M5 = 1, M6 = 1; M7 = 4), MDS/AML in 2 cases (M6 = 1; M7 = 1) and JMML in 1 case. FAB M6/M7 and transformed MDS were over-represented, reflecting the poor prognosis of these patients. The median white blood cell (WBC) count at diagnosis was 15.7 × 10^9^/L (1.5–38.0 × 10^9^/L). Cytogenetic analysis showed intermediate-risk AML in 8 cases and high-risk AML in 4 cases.

First-line CT was based on four different intensive regimens consisting of repeated courses of cytarabine plus intercalating agents. The patient with refractory JMML (Table 2, patient #1) received a combination of 6-mercaptopurine, etoposide, cytarabine, hydroxyurea and 6-thioguanine, plus 13 cis-retinoid acid, without responding. The two patients with primary refractory MDS/AML (patients #5 and #7) had received standard induction CT (cytarabine 200 mg/m^2^/d × 7 days plus mitoxantrone 12 mg/m^2^/d × 5 days), without responding. They were further treated with high-dose cytarabine and amsidine, but again no response was obtained. One of the two patients then received several courses of low-dose cytarabine plus etoposide before undergoing matched unrelated donor (MUD)-SCT while in partial remission. He relapsed 14 months later and was again refractory to low-dose cytarabine plus etoposide at the time of GO therapy.

The nine relapsing patients had been treated with FLAG (a combination of fludarabine, high-dose cytarabine and granulocyte colony-stimulating factor), [26] with or without anthracyclines. Eight of these nine patients (patients #2, #4, #6, #8, #9, #10, #11, #12) were in refractory reinduction CT before receiving GO. The ninth patient (UPN #3) entered CR2 after reinduction CT and underwent cord-blood SCT. This patient had no sign of active hepatic graft-versus-host disease or SOS and had normal transaminase and bilirubin values when GO was administered for very early post-SCT relapse.

The median interval between initial diagnosis and GO administration was 11.5 months (4.8–45.5 months). All patients had CD33+ myeloid leukemia at the time of GO therapy, with a median proportion of bone marrow blasts of 47% (20 – 98%). The median WBC count was 3.2 × 10^9^/L (0.2–22.0 × 10^9^/L). GO was given to 8 patients at a total dose of 9 mg/m^2^, administered in a single dose (n = 3) or divided into 3 doses given on days 1, 4 and 7 (n = 5). Patient #9 received 5 doses of 3 mg/m^2 ^(days 1, 4, 7, 28 and 31). Patient #10 received two doses of 9 mg/m^2 ^each (days 1 and 14). Patient #11 received two doses of 7.5 mg/m^2 ^each (days 1 and 16). Patient #12 received one dose of 6 mg/m^2 ^(day 1) and another dose of 9 mg/m^2 ^(day 16) (see Table 2). The unit doses and dosing intervals were based on those used in adults and in the pediatric studies of Sievers et al [22] and Zwaan et al [23], and were decided on by the physicians in charge of each patient. Fractionated doses were adopted secondarily, as they were reported to be associated with less SOS in adults (Raffoux E et al, annual meeting of the french hematologic society SFH, 2000, abstract). This schedule was chosen in the ongoing french MyloFrance protocol in adults.

Complete responses to GO were defined on the basis of the following consensus criteria: a bone marrow blast proportion of 5% or less, in the absence of leukemic cells in peripheral blood or elsewhere. The definition of CR included adequate recovery of peripheral blood cell counts (granulocytes > 1 × 10^9^/L and platelets > 100 × 10^9^/L with at least one week of transfusion independence). CRp was defined as a response with incomplete platelet recovery but with platelet transfusion independence [12]. Adverse events are reported according to the National Cancer Institute common toxicity criteria (NCI-CTC; 2003 revision) [27].

## Results

Responses and major toxicities are summarized in Table 2.

### Responses

Median follow-up after the beginning of GO treatment was 128 days (8–585 d). As shown in Table 2, responses were observed in 3 (25%) of the 12 patients. One patient entered CR on day 39, after 5 doses of 3 mg/m^2^, and two patients entered CRp, on day 28 after 3 doses of 3 mg/m^2 ^and on day 24 after 2 doses of 7.5 mg/m^2^. No change in bone marrow blast count was observed in two patients on day 15. Six patients progressed before day 15 of GO therapy. Patient #10 had paucicellular bone marrow, with few leukemic blasts, on day 66.

The three responding patients subsequently received SCT. Patient #5 received MUD-SCT 83 days after the first GO infusion and died on day 260 from infectious complications of SCT. Patient #9 received three GO doses of 3 mg/m^2^, which reduced the proportion of bone marrow blasts from 86% at baseline to 20% on day 28. Because GO was well tolerated, two more doses of 3 mg/m^2 ^GO were given, and CR2 was achieved on day 39. The patient relapsed 6.5 months after MUD-SCT. Further GO therapy controlled the bone marrow blast count, with no significant adverse effects, for 12.5 months after SCT. The patient finally died in blast crisis after a cumulative GO dose of approximately 45 mg/m^2^. Patient #11 received haplo-SCT 72 days after the first GO infusion. Bone marrow relapse occurred 9.6 months after SCT and the patient died 11.2 months after SCT. CR or CRp was maintained for 205, 260 and 288 days in the three responding patients.

Two patients (#4 and #6) who received three GO doses of 3 mg/m^2 ^(on days 1, 4 and 7) had no change in their bone marrow blast counts on day 15. They died of disease progression 97 and 101 days after the first GO infusion. Five patients (#1, #2, #3, #7 and #8) who progressed less than 15 days after GO treatment initiation developed grade IV hematologic toxicity related to the underlying leukemia. Patient #2 died from disease progression on day 8 after GO initiation. The remaining four patients received palliative therapy and died a median of 116 days after GO initiation. Patient #10, who did not respond to GO by day 66 (paucicellular bone marrow, with a few leukemic blasts) also developed grade IV hematologic toxicity probably related to the underlying disease. He died in blast crisis 238 days after GO initiation. Patient #12 did not respond by day 31, after two doses of GO (day 1 = 6 mg/m^2^, day 16 = 9 mg/m^2^). He then received alternative treatments and was alive in partial remission 120 days after GO administration. MUD-SCT was scheduled one month later.

Only one of the 12 patients was alive at the cutoff date for this analysis.

### Toxicity

In the three responding patients (#5, #9, #11), GO was well tolerated, with the exception of NCI-CTC grade III-IV hematologic toxicity. Patient #1 developed severe infusion-related hypotension (NCI-CTC grade IV) and fever, necessitating fluid support. Three other patients had febrile reactions during GO infusion (patients #7, #8 and #12). Five patients (#4, #7, #8, #11 and #12) had grade II vomiting during the infusion or the subsequent 24 hours. Two patients (#4 and #6) had grade II stomatitis, but the cause (GO or prolonged neutropenia?) was not determined. Four patients developed secondary infections: patient #7 had preseptic shock on day 12, but no bacterial or fungal pathogen was identified; patient #10 developed acute cholecystitis; and patients #3 and #4 had grade III febrile neutropenia with no identified site of infection. Patients #1 and #3 had transient NCI-CTC grade III transaminase elevation, accompanied by transient grade IV hyperbilirubinemia in one case (patient #1), but without ascites or weight gain suggestive of SOS. None of the 12 patients developed SOS.

## Discussion

We treated 12 children with relapsed or refractory myeloid leukemia with GO (3–18 mg/m^2^, up to 5 infusions) on a compassionate-use basis. The prognosis of such children is very poor: three of our patients with newly diagnosed JMML or MDS/AML were refractory to several different induction regimens; the remaining nine patients were either refractory to reinduction therapy or in second relapse after SCT. Further use of intensive CT is limited by its toxicity [28,29].

GO monotherapy thus yielded a response rate of 25% (3/12 patients). This rate is similar to that obtained in adults with relapsed AML [12,13]. However, available adult studies only included patients in first AML relapse. As in our series, the 15 children treated by Zwaan et al and the 29 children treated by Arceci et al with GO monotherapy had more advanced disease and, potentially, more cumulative toxicities [23,25]. Two CRp was followed by SCT in the Zwaan et al's series, and the two children were alive without significant GO/SCT-related toxicity, albeit with short follow-up (6 and 9 months). Likewise, our three patients who entered CR or CRp subsequently underwent SCT, and no major hepatotoxicity occurred. CR or CRp was maintained for 7 to 10 months before death or further relapse. In the dose-escalation study reported by Arceci et al, 4 patients experienced CR and 4 experienced CRp, for 8 (28%) overall remissions after GO therapy. Response rates were comparable in patients with refractory (30%) and relapsed (26%) disease. Most patients died of progressive disease (22/29), unfortunatly comprising 4/8 patients in CR or CRp after GO. These data suggest a need for intensive therapy following GO-induced remission, as in adults [30].

Considering the toxicity of GO, NCI-CTC grade IV hematologic toxicity occurred in all 12 children of our series. In responders, it was probably related to expression of the CD33 target by normal bone marrow progenitors. In non responders, it was difficult to determine the respective roles of GO and the underlying leukemia. With regard to non hematologic adverse events, GO was relatively well tolerated compared to intensive CT, with no cases of mucositis and only one noteworthy infection. This is in keeping with previous pediatric results [23,25]. GO therapy is an established risk factor for SOS [18-20]. No cases of SOS occurred in our series, even after SCT in the three responding patients. Zwaan et al reported only one case of SOS among 15 GO-treated children, occurring after SCT [23]. Arceci et al described an 24% overall incidence of SOS [25]. SOS developed in 6 of 7 patients after they underwent SCT. They had been exposed to GO within 3.5 to 4 months of transplantation. Two other patients who underwent SCT more than 4 months from GO exposure did not developed SOS. These findings are consistent with similar adult series [21]. Taken together, these data suggest that the time from treatment with GO to SCT for refractory/relapsed AML appears to be important with respect to the incidence of SOS after SCT. The incidence of liver dysfunction in GO-treated children is similar to that observed in the largest adult series [13], in which liver dysfunction was generally transient and never life-threatening.

GO was well tolerated, even during repeated infusions. One of our patients (#9) received a cumulative dose of approximately 45 mg/m^2 ^over a 14-month period. One patient in Zwaan's series was treated repeatedly with GO, with relatively long intervals between the infusions, and responded each time without showing signs of additional toxicity [23]. Palliative treatment thus appears to be feasible with repeated GO infusions.

## Conclusion

This report confirms that GO is clinically active in children with relapsed/refractory CD33+ AML. Despite its good tolerability, GO monotherapy only induces a response rate of 25 to 33%, similar to that previously reported in adults. In a recent trial, GO was combined with intensive CT as first-line treatment for AML in 72 patients aged 17 from 59 years [31]. Certain schedules of CT-GO combination induction therapy yielded CR in 91% of patients, 78% of whom were in continuous CR at 8 months. GO should be tested prospectively in a larger population of children with AML, with more stringent eligibility criteria and treatment schedules, in association with CT.

## Competing interests

The autor(s) declare that they have no competing interests.

## Authors' contributions

Conception and design: BB, AB, GL; analysis and interpretation of data: BB, AA, CG, KY, TL, YB, GL, AB; drafting the article or revising it critically for important intellectual content: AB, GL, BB, AA; final approval of the version to be published: all authors.

## Pre-publication history

The pre-publication history for this paper can be accessed here:



## Figures and Tables

**Table 1:** Characteristics of 12 children treated with GO.

*Nr*	*Age at diagnosis (yrs)*	*Gender*	*FAB*	*Cytogenetic Features*	*Molecular Biology*	*WBC at diagnosis (.10^9^/L)*	*WBC before GO (.10^9^/L)*	*Nb of Relapses*	*Treatment before relapse or GO*
#1	1.8	M	JMML	N	N	25.2	1.2	R	Other
#2	1.1	M	AML7	N	N	23.0	5.0	1	CT♣
#3	1.1	F	AML7	53,XX,+X,t(1;22)(p13;q13)+5,+6,+19,+20,+21 [18]	OTT/MAL	9.0	8.2	2	CT♣ IL2 CB-SCT
#4	14.0	F	AML1	47,XX,+8 [20]	N	27.7	2.2	1	CT♣
#5	14.7	M	MDS/AML6	N	N	1.5	0.9	R	CT♣ MUD-SCT
#6	17.2	F	AML2	46,XX,t(6;9)(p23;q34) [25]	DEK/CAN	3.3	10.9	1	CT♣
#7	2.8	M	MDS/AML7	46,XY,del(3)(q24;q26) [6]	N	11.4	22.0	R	CT♣
#8	2.5	M	AML7	N	N	4.8	7.4	1	CT♣
#9	1.0	F	AML7	46,t(2;16) [20]	N	20.0	4.0	1	CT♣
#10	8.2	M	AML5	46,XY,t(6;11)(q26;q23),del(12)(p11;p12) [17]	N	38.0	0.2	1	CT♣
#11	13.5	M	AML6	47,XY,del(3)(q23;qter), +8 [9]	N	2.6	1.2	1	Other
#12	10.0	F	AML2	45,XX,-7 [23]	N	26.0	2.4	1	CT♣

FAB indicates French-American-British classification; WBC, white blood cell; M, male; F, female; JMML, juvenile myelomonocytic leukemia; MDS, myelodysplasia; AML, acute myeloblastic leukemia; N, normal; R, refractory; CB-SCT, cord blood-stem cell transplantation; IL2, interleukine 2; MUD-SCT, matched unrelated donor-stem cell transplantation.

♣ CT, chemotherapy according to national contemporary protocols (LAME 91, LAME 99, ELAM 02, EORTC 58921)

**Table 2:** Modality and dose of GO therapy and treatment responses in 12 children with relapsed/refractory myeloid leukemia

*Nr*	*Disease status*	*Nb of GO courses*	*GO dose/course*	BM before GO % *	*Diag/GO (days)*	*Response*	*GO toxicity (NCI-CTC) ¥*	*Further treatment*	*Follow-up*
#1	De novo refractory	1	1 × 9 mg/m^2 ^(d1)	40	335	DP	Anaphylaxis Transient grade IV hyperbili. Grade III transa. elevation	None	V from JMML
#2	Refractory relapse	1	1 × 9 mg/m^2 ^(d1)	98	250	DP	No	None	V from AML
#3	Post-SCT 2^nd ^relapse	1	3 × 3 mg/m^2 ^(d1,d4,d7)	49	555	DP	Grade III febrile neutropenia Grade III transa. elevation	None	V from AML
#4	Refractory relapse	1	3 × 3 mg/m^2 ^(d1,d4,d7)	43	177	DP	Grade II vomiting Grade III febrile neutropenia Grade II stomatitis	None	V from AML
#5	De novo refractory	1	3 × 3 mg/m^2 ^(d1,d4,d7)	45	667	CRp (d28)	No	MUD-SCT	V from SCT
#6	Refractory relapse	1	3 × 3 mg/m^2 ^(d1,d4,d7)	73	579	DP	Grade II stomatitis	None	V from AML
#7	De novo refractory	1	1 × 9 mg/m^2 ^(d1)	59	145	DP	Septic shock Grade II vomiting Grade I fever	None	V from AML
#8	Refractory relapse	1	3 × 3 mg/m^2 ^(d1,d4,d7)	96	298	DP	Grade II vomiting Grade I fever	None	V from AML
#9	Refractory relapse	2	3 × 3 mg/m^2 ^(d1,d4,d7) and 2 × 3 mg/m^2 ^‡	86	162	CR (d39)	No	MUD-SCT	V from AML §
#10	Refractory relapse	1	2 × 9 mg/m^2 ^(d1,d14)	25	357	DP	Grade III febrile neutropenia Grade II transa elevation Grade II hyperbilirubinemia Grade III acute cholecystitis Grade IV hemorragic cystitis	None	V from AML
#11	Refractory relapse	1	2 × 7.5 mg/m^2 ^(d1,d16)	20	513	CRp (d24)	Grade II fever Grade II vomiting	Haplo-SCT	V from AML
#12	Refractory relapse	1	6 mg/m^2 ^(d1)+9 mg/m^2 ^(d16)	35	1367	DP	Grade II pancreatitis Grade II fever Grade II vomiting	Arsenic clofarabin.	Alive in PR ♣

BM: bone marrow; SCT, stem cell transplantation; MUD, matched unrelated donor; CR, complete remission; CRp, complete remission without total platelet recovery; PR, partial remission; DP, disease progression.

*Bone marrow blast percentage. CD33 expression was greater than 20% before GO in all cases.

¥All patients experienced NCI-CTC grade III to IV hematological toxicity, which is not mentioned here.

‡This patient was treated with 3 × 3 mg/m^2 ^of GO leading to a reduction in BM blasts from 86 to 20% at d28. Because of good tolerance, two more doses of 3 mg/m^2 ^were administered and CR was obtained at d39.

§This patient relapsed 69 days after MUD-SCT but a drastic reduction of BM blasts was obtained with 5 weekly doses of 3 mg/m^2 ^GO without any significant adverse effects. He received GO maintenance therapy but died 12 months after MUD-SCT.

V Death

♣ This patient was scheduled to receive MUD-SCT.
